# *k*-Space tutorial: an MRI educational tool for a better understanding of *k*-space

**DOI:** 10.2349/biij.4.1.e15

**Published:** 2008-01-01

**Authors:** D Moratal, A Vallés-Luch, L Martí-Bonmatí, ME Brummer

**Affiliations:** 1 Electronics Engineering Department, Universitat Politècnica de València, Valencia, Spain; 2 Applied Thermodynamics Department, Universitat Politècnica de València, Valencia, Spain; 3 Radiology Department, Hospital Universitari Dr. Peset, Valencia, Spain; 4 Radiology Department, Hospital Quirón, Valencia, Spain; 5 Pediatrics and Radiology Departments, Emory University School of Medicine, Atlanta, Georgia, United States of America

**Keywords:** Magnetic resonance imaging, tutorial, *k*-space, artifacts

## Abstract

A main difference between Magnetic Resonance (MR) imaging and other medical imaging modalities is the control over the data acquisition and how it can be managed to finally show the adequate reconstructed image. With some basic programming adjustments, the user can modify the spatial resolution, field of view (FOV), image contrast, acquisition velocity, artifacts and so many other parameters that will contribute to form the final image. The main character and agent of all this control is called k-space, which represents the matrix where the MR data will be stored previously to a Fourier transformation to obtain the desired image.

This work introduces 'k-Space tutorial', a MATLAB-based educational environment to learn how the image and the *k*-space are related, and how the image can be affected through *k*-space modifications. This MR imaging educational environment has learning facilities on the basic acceleration strategies that can be encountered in almost all MR scanners: scan percentage, rectangular FOV and partial Fourier imaging. It also permits one to apply low- and high-pass filtering to the *k*-space, and to observe how the contrast or the details are selected in the reconstructed image. It also allows one to modify the signal-to-noise ratio of the acquisition and create some artifacts on the image as a simulated movement of the patient – with variable intensity level – and some electromagnetic spikes on *k*-space occurring during data acquisition.

## INTRODUCTION

In magnetic resonance (MR) imaging the user has control in many ways over how the data are acquired and how they can be manipulated to influence the reconstructed image. The technician can modify parameters affecting the spatial and temporal resolution, the field of view, the contrast, the speed of the acquisition and the influence of various types of artifacts. This is possible due to what is known as *k*-space, the data matrix obtained directly from the magnetic resonance (MR) scanner before any kind of processing and the Fourier transform application, which will provide the final reconstructed image [1,5].

In this work a '*k*-Space tutorial' is introduced, where the technicians, residents in radiology and medical imaging students will be able to analyse the influence of *k*-space on the reconstructed image, simulating different types of filtering, three basic acceleration strategies and different types of simulated artifacts to observe changes on the spatial resolution, field of view or signal-to-noise ratio (SNR) of the associated image.

### Raw data

Raw data in MRI are the transversal components of the magnetisation in the imaging object after excitation, sampled from the receiver coil signal and stored as a function of time during the data acquisition of an MR imaging sequence. In a transverse slice, the horizontal axis is usually set as the frequency encoding direction, while the vertical axis is the phase encoding direction of excited protons. This is also known as *k*-space data.

### k-Space

The *k*-space is an extension of the concept of Fourier space well known in MR imaging. The *k*-space represents the spatial frequency information in two or three dimensions of an object. The *k*-space is defined by the space covered by the phase and frequency encoding data.

The relationship between *k*-space data and image data is the Fourier transformation. The data acquisition matrix contains raw data before image processing. In 2-dimensional (2D) Fourier transform imaging, a line of data corresponds to the digitised MR signal at a particular phase encoding level. The position in *k*-space is directly related to the gradient across the object being imaged. By changing the gradient over time, the *k*-space data are sampled in a trajectory through Fourier space.

Every point in the raw data matrix contains part of the information for the complete image. A point in the raw data matrix does not correspond to a point in the image matrix. The outer rows of the raw data matrix, the high spatial frequencies, provide information regarding the borders and contours of the image, the detail of the structures. The inner rows of the matrix, the low spatial frequencies, provide information on the general contrast of the image [1-5].

## MATERIALS AND METHODS

**Figure 1:**
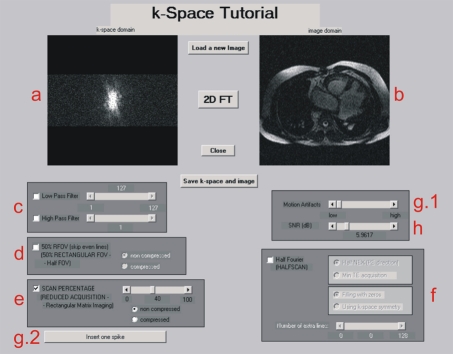
Main window of the *k*-Space tutorial. At the upper part of the window we can see the *k*-Space (a) and its associated image (b) to which all the desired operations will be applied. After having applied the desired operations (c-h), it is also possible to save the resulting image and its *k*-Space

This educational tool, called '*k*-Space tutorial', has been developed using MATLAB R2006a (The Mathworks, Inc, Natick, MA) implementing a visual and easy-to-use Graphical User Interface, as can be observed in Figure 1. With this tutorial the user will be able to apply a low-, high- and band-pass filtering to the *k*-space as well as study the basic acceleration strategies to reduce scan time and observe how the image is affected: rectangular field-of-view, sampling truncation and partial Fourier imaging, without using any kind of filtering. In addition to this, the signal-to-noise ratio of the image can be varied adding a white Gaussian noise to the *k*-space, and some motion-related and electromagnetic artifacts can be simulated on the *k*-space observing their influence on the associated image.

All the experiments performed in this study were done on a cardiac MR transversal slice image of a patient with transposition of great vessels. This image was acquired using a steady state gradient echo sequence (balanced Fast Field Echo) with a 256 x 256 image grid from 192 phase-encodings. Nevertheless, this *k*-space tutorial permits one to load any image and apply every parameter or function of this educational environment to any image selected by the user either in DICOM (digital imaging and communications in medicine) or BMP (bitmap) format.

## RESULTS

### k-Space filtering

**Figure 2:**
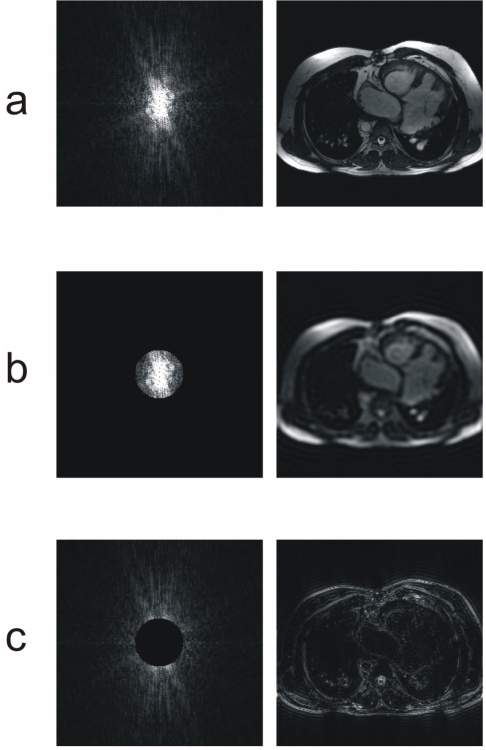
*k*-Space (left) and its associated image (right). (a) Original *k*-Space and its associated image. (b) Low pass filtering on the original *k*-Space. The resulting image only shows the contrast of the image. The information of the high spatial frequencies, that contains the details and contours of the objects, have disappeared. (c) High pass filtering, where only the high spatial frequencies have been selected in the *k*-Space, providing only information about the details and edges of the objects in the image domain.

Figure 2a shows the image and its associated *k*-space over which all the detailed operations of this educational environment will be applied. With this tool the user can apply a low-pass filtering to the *k*-space and see its influence over the image (figure 2b). After this low-pass filtering, the contrast of the image is maintained but the details and contours of the objects disappear. This is due to the low spatial frequencies that have been selected, eliminating the high spatial frequencies.

Similarly, the user can apply a high-pass filtering to the *k*-space and see that only the contours and details of the objects of the image have been selected, as depicted in Figure 2c.

The user has also the possibility of combining a low- and high-pass filtering, producing a band-pass filtering.

### Field of view and sampling interval

With this tool, one can evaluate the three basic acceleration strategies encountered nowadays in any MR scanner. These reduced sampling techniques are the rectangular field of view (RFOV), reduced scan percentage and partial Fourier imaging.

**Figure 3:**
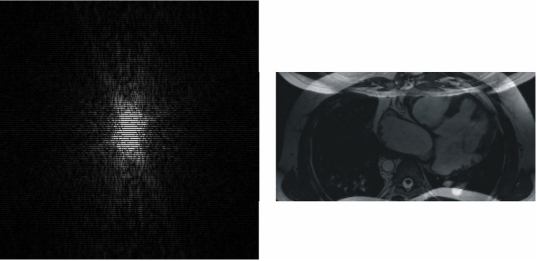
Rectangular field of view at 50%. The spacing between phase-encodings (vertical direction) in the *k*-space produces a reduction of the FOV in the image in the same proportion and in the same direction (it does not affect the FOV size in the other direction).

The RFOV technique acquires a different scanned region in the phase encoding directions. It means that the data is acquired with fewer measurement lines, thus reducing the scan time. Because there are fewer rows than columns, a rectangular image is obtained, providing the name to this technique. Reducing the FOV in the phase encoding direction saves scan time by decreasing SNR but invariably maintains the spatial resolution. If the object is larger in this phase direction than the RFOV, a foldover artifact will appear. As can be observed in Figure 3, the spacing between the acquired phase-encodings has been doubled, and then the associated FOV has been reduced in the same proportion, thus producing a foldover in the phase-encoding direction.

### Sampling truncation (scan percentage)

**Figure 4:**
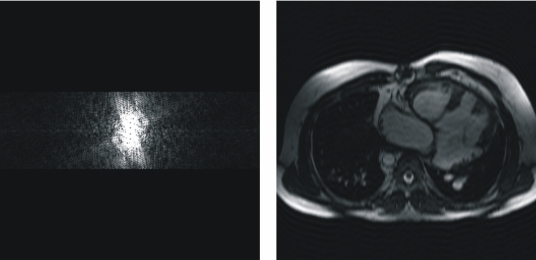
Scan percentage at 30%. Only 30% of the central data of *k*-space has been acquired, resulting in a reduction of the spatial resolution in the image as well as in an increase of the SNR in the image.

Reducing the sampling truncation (scan percentage) to less than 100% means not acquiring the most peripheral lines in *k*-space, thus affecting the image resolution, the SNR and the scan time of the acquisition. By reducing the scan percentage the resolution decreases but the SNR increases while the scan time is reduced in the same proportion because fewer phase-encodings must be acquired. Figure 4 shows a scan percentage of 30% simulated with the developed tool. The omission of so many high spatial frequencies means that many edges and much of the fine detail will be lost. In addition to this, some truncation artifacts can be seen in the same direction in which the reduction of data acquisition has been implemented because the high contrast boundary cannot be properly represented without the higher spatial frequencies. A sampling truncation of 80% will usually have little discernable effect on the image.

With this tool, one can easily evaluate the reduction of the spatial resolution suffered by the associated image as the scan percentage parameter value decreases (the number of acquired phase-encodings gets smaller).

### Half (partial) Fourier imaging

The third basic acceleration strategy that can be studied using this *k*-space tutorial is called half (or partial) Fourier imaging. This technique consists of reconstructing an image from an MR data set comprising an asymmetric sampling of *k*-space. This can be done because of the phase conjugate symmetry benefits from the Hermitian symmetry of the raw data in *k*-space. It can be used either to shorten image acquisition time, by reducing the number of required phase encoding steps, or to shorten the echo time (TE) by moving off-center the echo in the acquisition window. In either case the signal-to-noise ratio is reduced and the resolution can be improved to the maximum available data resolution.

**Figure 5:**
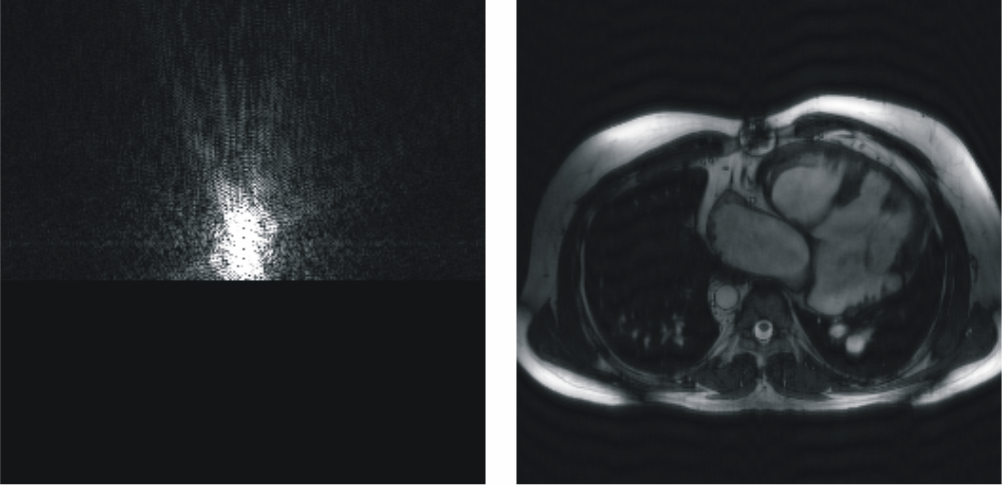
Half (partial) Fourier imaging in the phase-encoding direction (vertical direction), reducing the number of phase-encoding steps necessary to reconstruct the image, and thus reducing the scan time. The non-acquired phase-encodings are filled with complex conjugate data of the other half plane. In the example shown in this figure, 20 additional phase-encodings have been acquired in the center of *k*-space for phase-coherence.

Figure 5 shows an example of half Fourier imaging to shorten acquisition time in the phase-encoding direction, without the use of any kind of filtering. Note that some extra-lines need to be acquired for phase-coherence. This number of extra lines can be selected by the user, studying also the influence of this parameter on the reconstructed image. The non-acquired phase-encodings are filled with complex conjugate data of the other half plane.

### Signal-to-noise ratio (SNR)

The SNR is a criterion of image quality. This parameter describes the relative contributions to a detected signal of the true signal and random superimposed background noise signals.

**Figure 6:**
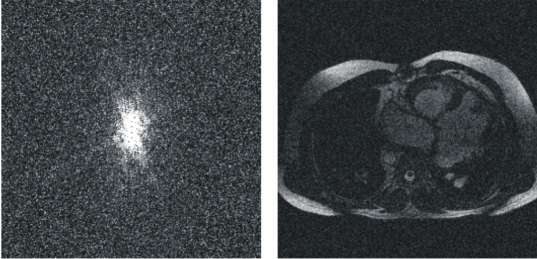
*k*-Space and its reconstructed image where the SNR has been adjusted to 4.04 dB.

With the *k*-Space tutorial tool, the user can modify the SNR of the reconstructed image as can be observed in Figure 6.

### Artifacts

An MR image artifact is a structure not normally present but visible as a result of the complex interaction of contemporary imager subsystems, or in other cases as a consequence of environmental influences as heat or humidity or caused by the human body. An understanding of the sources of artifacts enables optimisation of the MR imaging system performance, finally improving the quality of the acquired images [6].

Artifacts may be very noticeable or just a few pixels out of balance, but can give confusing non-real appearances that may be misdiagnosed as disease or hidden lesions. Many types of artifacts may occur in MR imaging and they are typically classified according to their basic principles, e.g.:

- Physiologic (motion, flow)
- Hardware (electromagnetic spikes, RF pulse truncation, RF interference)
- Inherent physics (chemical shift, susceptibility, metal)

### Motion-related artifacts

Patient motion during image acquisition is one of the main sources of artifacts. They appear as a blurring of the image as well as ghosting in the phase encoding direction. The time difference in the acquisition of adjacent points in the frequency encoding direction is relatively short, in the order of microseconds, and is dependent on the sampling frequency or the bandwidth used. The time difference in the acquisition of adjacent points in the phase encoding direction is much longer and is equal to the repetition time (TR) used for the sequence acquisition. The positional difference because of motion introduces a phase difference between the views in *k*-space that appears as a ghost on the image. Respiratory and cardiac motions are frequent causes of movement-related artifacts in the phase encoding direction [6].

**Figure 7:**
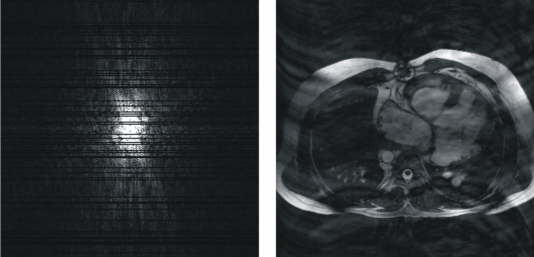
Motion-related artifact, due to a severe movement of the patient, simulated using the presented *k*-Space tutorial.

Using the introduced *k*-Space tutorial the user can simulate a gradually motion-related artifact of the patient from a light to a severe movement (see Figure 7) taking out a specific number of randomly selected phase-encodings. This software does not simulate any phase-shift on the data.

### Criss-cross/herringbone (electromagnetic spikes) artifacts

**Figure 8:**
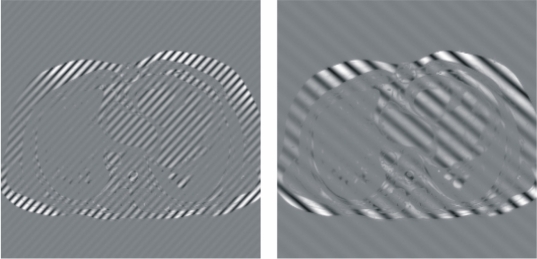
Bad data points in *k*-space result in band artifacts on the MR image. The location of the bad data points, and their distance from the center of *k*-space, determine the angulation of the bands and the distance between them. The intensity of the spike determines the severity of the artifact. The displacement of the spike of noise from the center of *k*-space determines the spacing between the stripes and the angulation of the stripes with respect to the readout direction. The displayed images show the resulting images from two different *k*-spaces where a spike has been simulated using the '*k*-Space tutorial'. The stripes shown in the image on the left are closer to those shown in the image on the right, indicating that the spike has occured further to the centre of its corresponding *k*-space (higher spatial frequency) than that of the image on the right. Following the angulation of the bands of the images, it can be known that the spike of the associated *k*-space of the image on the left has occured in the upper-left or in the lower-right quadrant of the *k*-space while the spike of the associated *k*-space of the image on the right has occured in the upper-right or in the lower-left quadrant of its corresponding *k*-space.

Gradients applied at a very high duty cycle (e.g., those in echo-planar imaging), electronic equipment inside the MR procedure room, or fluctuating alternating current may produce bad data points, or a spike of noise, in the *k*-space. This bad data might be a single point or a few points in *k*-space that have a very high or low intensity compared with the intensity of the rest of *k*-space. The convolution of this spike, that can be simulated using the '*k*-Space tutorial', with all the other image information during the Fourier transform results in dark stripes overlaid on the image [6] (see Figure 8).

## DISCUSSION AND CONCLUSION

This paper presented an educational tool for a better understanding of the influence of the *k*-space characteristics on the final reconstructed image. This tool permits the user to play with the most important parameters of the *k*-space that can influence the corresponding image, to understand how the spatial resolution, field of view or scan time of an image are completely controlled from its *k*-space. It also permits the user to simulate some motion-related artifacts as well as electromagnetic spikes on the image, permitting the user to better understand the concept of *k*-space spatial frequencies.

This tool has been tested by residents in radiology and engineering students of medical imaging courses, achieving a high level of satisfaction and accomplishing its main objective: to help to better understand the sometimes unknown and always difficult to comprehend the concept of *k*-space in MR imaging and how the image is inherently associated to it. This tool is freely available upon request to the corresponding author.
